# Impact of the COVID-19 Pandemic on Children and Adolescents with New-Onset Type 1 Diabetes

**DOI:** 10.1155/2023/7660985

**Published:** 2023-05-30

**Authors:** Clemens Kamrath, Alexander J. Eckert, Reinhard W. Holl, Joachim Rosenbauer

**Affiliations:** ^1^Centre of Child and Adolescent Medicine, Justus Liebig University, Giessen, Germany; ^2^Institute of Epidemiology and Medical Biometry, ZIBMT, Ulm University, Ulm, Germany; ^3^German Centre for Diabetes Research (DZD), Munich-Neuherberg, Munich, Germany; ^4^Institute for Biometrics and Epidemiology, German Diabetes Centre, Leibniz Centre for Diabetes Research at Heinrich Heine University Dusseldorf, Dusseldorf, Germany

## Abstract

**Background:**

The COVID-19 pandemic has an impact on the incidence of type 1 diabetes and frequency of diabetic ketoacidosis. However, the exact relationships are unclear. It is also not known whether this is a short-term phenomenon or whether the effects have long-term relevance. Furthermore, it is not known whether these changes during the pandemic are due to direct effects of SARS-CoV-2 or to changes in the patient's environment during the pandemic.

**Methods:**

We conducted an extensive literature search on PubMed. For the estimation of relative risks of new-onset type 1 diabetes, we applied a Poisson regression model and for the comparison of incidences and we included the logarithm of person-years. Furthermore, we performed a meta-analysis using the logarithm of the relative risk for new-onset type 1 diabetes as effect size.

**Results:**

Pooling the relative risk estimates in a random-effects meta-analysis revealed that the type 1 diabetes incidence rate increased by 20% (relative risk 1.200 (95% CI 1.125, 1.281)), and that the risk of new-onset type 1 diabetes after a SARS-CoV-2 infection increased by 62% (relative risk 1.622 (95% CI 1.347, 1.953)) compared with the prepandemic period.

**Conclusion:**

There is considerable evidence that there is an increase in type 1 diabetes in children during the COVID-19 pandemic. Many studies suggesting a direct effect of SARS-CoV-2 have methodological weaknesses. As no evidence of an increase in presymptomatic cases with isolated islet autoimmunity was found, this could also suggest an accelerated transition from presymptomatic patients to clinically overt type 1 diabetes. Furthermore, there was a marked exacerbation of the preexisting increase in the prevalence of diabetic ketoacidosis at diagnosis of type 1 diabetes during the pandemic. Both the increased incidence of paediatric type 1 diabetes and the higher prevalence of diabetic ketoacidosis at diagnosis led to a massive rise in the number of children with diabetic ketoacidosis during the pandemic.

## 1. Introduction

Genetic and environmental factors such as infections are relevant for the development of type 1 diabetes (T1D) [1–6]. Therefore, early after the occurrence of the severe acute respiratory syndrome coronavirus 2 (SARS-CoV-2), the virus leading to the coronavirus disease 2019 (COVID-19) pandemic, it has been suggested that there might be an association with the incidence of T1D [7, 8].

It has been argued that the angiotensin-converting enzyme 2 (ACE2) receptor and other coreceptors serve SARS-CoV-2 viruses as cell entry ports and that these are also expressed in insulin-producing *ß*-cells of the pancreas [9–12]. Various mechanisms have been considered for the potentially diabetogenic effect of SARS-CoV-2 infection. Besides direct *ß*-cell damage or direct effects on immune regulation with triggering or acceleration of an autoimmune process, indirect effects of the pandemic have also been discussed [13–16]. Measures to control the COVID-19 pandemic led to a decrease in common paediatric viral infections but also to increased psychological stress in children and adolescents and, as a consequence, possibly to an increased incidence of T1D [17–20]. However, the observed change in the number of new cases of T1D in the pandemic is very heterogeneous between studies.

## 2. Methods

### 2.1. Literature Search Strategy

Starting point of the search for our narrative review was the recent meta-analysis by Rahmati et al. on the impact of the COVID-19 pandemic on the incidence and presentation at onset of paediatric T1D comprising studies that compared new-onset T1D cases and T1D incidence in a pandemic and a prepandemic period [21]. We additionally searched PubMed for more recent publication reporting respective data. Furthermore, we searched for studies comparing the T1D risk in cohorts with documented SARS-CoV-2 infection compared to cohorts without documented SARS-CoV-2 infection or with other respiratory infections. Search terms used included “type 1 diabetes,” “diabetes mellitus,” “diabetes onset,” “new-onset cases,” “incident cases,” “incidence,” “diabetic ketoacidosis,” “severe diabetic ketoacidosis,” “acidosis,” “COVID-19,” “pandemic,” and “SARS-CoV-2 infection.” Finally, we searched the reference list of all selected studies.

### 2.2. Eligibility Criteria and Extraction of Data

We included studies that allowed to compare the frequency of T1D (number of new-onset T1D cases or T1D incidence) in a pandemic with a prepandemic period or that compared the risk of T1D in cohorts with documented SARS-CoV-2 infection and cohorts without SARS-CoV-2 infection or other respiratory infections whereby comparison cohorts included contemporary pandemic cohorts and historical prepandemic cohorts.

From these studies, we extracted region of study, data source (single-centre, multicentre, and electronic health record database), age range comprised, the pandemic and prepandemic period observed, and the respective numbers of paediatric new-onset T1D cases as well as T1D (cumulative) incidences, if reported. Furthermore, we extracted an estimate of the T1D risk increase as relative risk (ratio of incident cases, incidence rate ratio, or hazard ratio) with the respective 95% confidence interval (CI), if reported, during the pandemic period compared to prepandemic periods or in cohorts with SARS-CoV-2 infection compared to cohorts without SARS-CoV-2 infection.

From the meta-analysis by Rahmati et al. [21], we only extracted pooled data for the comparison of incident cases in the pandemic and the prepandemic period and included this data in the meta-analysis. Individual data of the studies underlying this pooled data were not extracted and not considered.

With regard to the risk of diabetic ketoacidosis (DKA) during the COVID-19 pandemic, there are already recent meta-analyses by Alfayez et al. [22] and a systematic review about DKA risk factors by Rugg–Gunn and colleagues [23]. Therefore, we refrained from a new meta-analysis but instead present additional existing evidence and new studies.

### 2.3. Statistical Methods

In case of differences between the length of pandemic and prepandemic observation periods, we estimated the expected number of T1D cases for the pandemic period from the observed number of cases in the prepandemic period accounting for the different length of observation periods. Furthermore, if single figures were missing (e.g., relative risk or expected number of pandemic cases), we estimated these from the data reported (e.g., observed and expected pandemic cases/incidences or from expected number of pandemic cases and a respective relative risk estimate), as far as possible.

For the estimation of relative risks, we applied a Poisson regression model with the number of cases as dependent variable and a variable indicating observed and expected data for the pandemic period as independent variable. In case of comparison of incidences, the logarithm of person-years was included as off-set.

We performed the meta-analysis using the logarithm of the relative risk for T1D as effect size. Depending on the design of individual studies, the relative risk corresponds to the ratio of incident cases or the incidence rate in the pandemic compared to the prepandemic period, or the incidence rate ratio or hazard ratio comparing pandemic cohorts with SARS-CoV-2 infection and pandemic/prepandemic cohorts without SARS-CoV-2 infection/with other respiratory infection. To pool the relative risk estimates of included studies we applied the DerSimonian–Laird random-effects model [24]. Results are presented as relative risks with 95% CI in a forest plot.

All analysis were performed using SAS version 9.4 (2016; SAS Institute Inc., Cary, North Carolina) or Stata version 17 (StataCorp. 2021. Stata: Release 17. Statistical Software. College Station, TX: StataCorp LLC).

## 3. Increase in Cases with New-Onset Type 1 Diabetes in Children during the COVID-19 Pandemic

A recent meta-analysis based on 24 studies found an 8.3% (95% CI 2.8, 14.2; *p*=0.003) increase in new cases in the 2020 pandemic year compared with 2019 [21]. However, the changes in T1D incidence observed in the individual studies during the COVID-19 pandemic were very heterogeneous, ranging from a 43.3% decrease in new cases to a 100% increase [7, 25].

**Figure 1:**
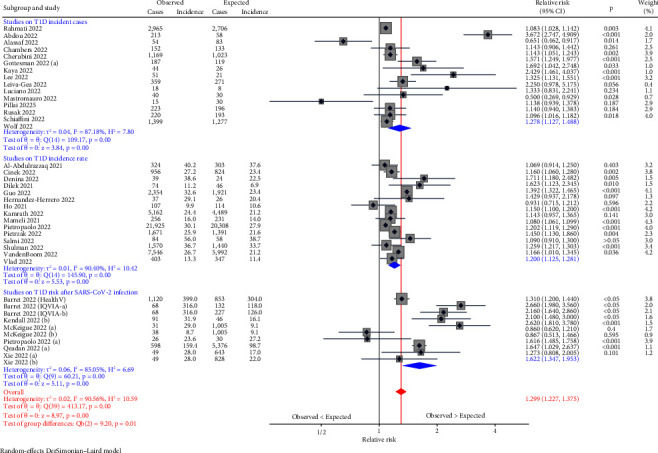
Random-effects meta-analysis of the risk of type 1 diabetes in the pandemic vs. the prepandemic period or in pandemic cohorts with COVID-19 vs. pandemic or prepandemic cohorts without COVID-19 or other respiratory infections. *τ*^2^: between-study variance, *H*^2^: statistic of between-study heterogeneity (=1/(1−I2)), *Q*, *p*: Cochran's *Q* statistic for testing heterogeneity between studies and corresponding *p* value, *z*, *p*: *z*-statistic for testing the hypothesis of a zero pooled effect and corresponding *p* value, and Q_b_, *p*: test statistic for testing between group differences of the pooled effect and corresponding *p* value *I^2^*: statistic of between study heterogeneity.

Even in more recent studies [26–39], the observed changes in the number of new cases between the pandemic and the prepandemic period were very inconsistent, ranging from a 50% decrease to a 267% increase (Table 1, Figure 1). Three large multicentre studies found an increase between 10% and 33% [29, 33, 39].

All of these studies had the disadvantage that the long-term trend in the number of new cases (with the exception of [30]) and changes in the size of the background population and thus in person-years under risk were not taken into account. Furthermore, SARS-CoV-2 infection was detected in only a small proportion of new T1D cases in some studies, suggesting that the increase in new T1D cases is probably not directly attributable to SARS-CoV-2 infections.

### 3.1. Results of Meta-Analysis

Pooling of relative risk estimates in a random-effects meta-analysis showed that the number of cases with new-onset T1D in the pandemic period increased by 28% (relative risk 1.278 (95% CI 1.127, 1.448)) compared to the prepandemic period (Figure 1).

## 4. Increase in the Incidence Rate of Type 1 Diabetes in Children during the COVID-19 Pandemic

Studies on the incidence of T1D in childhood considering the background risk population also showed very different trends of the incidence rate of T1D during the COVID-19 pandemic compared to previous years [16, 40–53]. Changes ranged from a 7% decrease to a 71% increase. The ten largest studies reported an increase in the incidence rate between 7% and 33% during the COVID-19 pandemic (Table 2, Figure 1).

According to a German nationwide study, the observed incidence rate in the pandemic period from January 2020 to June 2021 was 15% (10, 20%) higher than expected based on the long-term trend of previous years (24.4 vs. 21.2 per 100,000 person-years) [16]. Comparably, higher incidence rates were seen in boys and girls. A higher incidence rate increase of 23% (13, 33%) was observed in under 6-year-olds, whereas a nonsignificant lower increase of 6% (−2, 13%) was observed in 12 to 17-year-olds. Interestingly, incidence rates were significantly higher than expected only in the two periods from June to September 2020 and from March to June 2021 (24.0 vs. 18.9 and 25.5 vs. 20.2 per 100,000 person-years, respectively; increase of 27% each). Month-specific analyses revealed significantly higher than expected incidence rates in June and July 2020 (increase 43% and 48%, respectively) and in March and June 2021 (incidence rate increase 29–48%). Accordingly, an increased incidence rate was observed about 3 months after the respective peaks of the first three pandemic waves. A Finnish study also showed a comparable time lag of the increase in incidence rate in the COVID-19 pandemic [50]. Information on SARS-CoV-2 infection in the period before manifestation of T1D was not available in the German study; so, the direct impact of SARS-CoV-2 infection on the incidence rate of new cases could not be investigated. Consistent with a previous analysis [54], the proportion of autoantibody-negative T1D did not change in the extended pandemic period from January 2020 to July 2021 compared with the previous two years [16].

The increase in the incidence of T1D during the COVID-19 pandemic was confirmed by another study from Germany [52]. This study analysed the weekly incidence of T1D in children and adolescents under 20 years of age during the COVID-19 pandemic from March 2020 to December 2021, based on new insulin prescriptions from 2016 to 2021 using the IQVIA longitudinal prescription database (LRx). The observed incidence of T1D, defined by new insulin prescriptions, was 25.9% (21.7, 30.3) higher than expected during the 96 weeks from March 2020 to December 2021. In addition, during the summer of 2020 and 2021, T1D incidence was even 44% and 65% higher, respectively, than expected. Furthermore, no cross-correlation between the incidences of COVID-19 and T1D was found in this study, and thus no evidence that the increase in T1D incidence during the pandemic was related to direct effects of SARS-CoV-2.

In Canada, increased incidence rates were found in comparable months as in Germany. Using health insurance data for the pandemic period from March 2020 to September 2021, a study in Ontario found a nonsignificant 9% (−9, 30%) higher incidence rate of T1D than expected [51]. At the beginning of the pandemic, from March to May 2020, the incidence rate was 15% to 32% lower than expected; in June and July 2020, the incidence rate was 12% (not significant) and 27%, respectively, and from February to July 2021, the incidence rate was 33% to 50% (significant) higher than expected. According to the authors, this progression could be explained by a possible delay in diagnosis and subsequent catch-up effects. Considering the usual period of only a few weeks from first symptoms to diagnosis of T1D, this explanation can certainly only apply to the increase in 2020. One drawback of the study is that it did not distinguish between type 1 and type 2 diabetes; but typically, 95% of children and adolescents with diabetes in Ontario have T1D.

Very similar to the studies from Germany and Canada, the observed incidence rate of T1D in children in Czechia in 2020 and 2021 was 27.2 per 100,000 person-years, 16% (6, 28%) higher than trends in the prepandemic years 2010 to 2019 would have suggested [41]. As shown in Ontario, the T1D incidence rate had a trough during the first lockdown from March to May 2020 and then it rose above expected values forecasted from the incidence rate time series between 2010 and 2019, a pattern that continued almost for 19 months of observation.

### 4.1. Results of Meta-Analysis

Pooling of relative risk estimates in a random-effects meta-analysis showed that the T1D incidence rate in the pandemic period increased by 20% (relative risk 1.200 (95% CI 1.125, 1.281)) compared to the prepandemic period (Figure 1).

## 5. Risk for Type 1 Diabetes after COVID-19

An increased risk of developing T1D after COVID-19 cannot be ruled out. Six recent studies investigated whether individuals with SARS-CoV-2 infection are at increased risk of developing (type 1) diabetes using large health service data sets (Table 3, Figure 1) [48, 55–59].

A study of IQVIA data in the USA found a 166% (98, 256%) higher incidence rate of diabetes (type 1, type 2, or other diabetes) in children and adolescents with documented SARS-CoV-2 infection at least 30 days before diabetes diagnosis [55]. The incidence rate more than 30 days after COVID-19 was 116% (64, 186%) higher compared to children and adolescents with documented acute respiratory infections not due to COVID-19 before to the pandemic. The analogous analysis of HealthVerity data found a 31% (20, 44%) higher incidence rate following COVID-19. Methodological weaknesses are that the study did not distinguish between type 1 and type 2 diabetes and that asymptomatically SARS-CoV-2-infected children (which is quite frequent) may have been misclassified as having no infection. Infected and not infected persons were the two risk populations in which diabetes cases were observed. If many asymptomatically infected children were misclassified as not infected, person-years under risk will decrease for infected persons and increase for those not infected; i.e., incidence rate will be overestimated for infected persons and underestimated for not infected persons. As a result, this could have led to an overestimation of the risk of diabetes after COVID-19 due to a high prevalence of children with asymptomatic SARS-CoV-2 infections [60].

A further study using similar methodology from Scotland in a cohort of 1,849,411 people under 35 years of age found a 162% (81, 278%) higher incidence rate of T1D in children and young adults with confirmed COVID-19 in the past 30 days [56]. In those aged 0–14 years, incidence rate of T1D during 2020-2021 was 20% higher than the 7-year average. Despite this significantly increased risk, the authors suggested that SARS-CoV-2 infection itself was unlikely to be the cause of this increase and therefore did not assume a causal relationship between SARS-CoV-2 infection and the occurrence of diabetes. Reasons given for the absence of such an association were an increased SARS-CoV-2 test rate at the time of clinical onset of diabetes, a possible diabetes onset already before the positive SARS-CoV-2 test result, and a lack of temporal congruence of T1D and COVID-19 incidence rates. The authors also suggested several possible causes other than SARS-CoV-2 infections for the higher T1D incidence rate during the pandemic compared to the long-term mean, such as altered levels respiratory virus or enteroviruses infections or changes in other environmental factors during the pandemic that are associated with altered T1D risk.

Using an analysis of 27,292,879 patients from the Cerner Real-World-Data, Qeadan and colleagues found a significant 42% increase (38, 46%) in the risk of developing T1D within two months of a SARS-CoV-2 infection compared with people without COVID-19 [57]. There was an increased risk in the paediatric age groups: 0–1 years odds ratio (OR) 6.8 (95% CI 2.8, 17.0), 2–5 years (OR 2.2 [1.7, 2.9]), 6–12 years (OR 2.0 [1.8, 2.3]), and 13–17 years (OR 1.6 [1.4, 1.8]). However, classification bias would be possible if patients with T1D or SARS-CoV-2 infection were not correctly identified.

Using a web-based database of electronic health records of more than 90 million patients, Kendall et al. reported that new T1D diagnoses were more likely in paediatric patients with previous COVID-19 than in patients with other respiratory infections [58]. The study compared the risk of new diagnosis of T1D in 285,628 children with SARS-CoV-2 infection between March 2020 and December 2021 with 285,628 matched children with respiratory infections not due to SARS-CoV-2. At 6 months, 123 patients (0.043%) had developed T1D after COVID-19 compared with 72 patients (0.025%) after non-COVID-19 respiratory infections, according to a hazard ratio of 1.83 (1.36, 2.44).

A further study surveyed the TriNetX COVID-19 research network, a global federated electronic medical records network containing data of more than 80 million patients from 60 healthcare organizations [48]. Patients with a COVID-19 diagnosis were identified by positive SARS-CoV-2 PCR tests or by ICD-10 diagnosis codes. Among those up to 18 years of age, the incidence of insulin-dependent diabetes was statistically indistinguishable in patients with previously diagnosed and confirmed COVID-19 from the control population without COVID-19.

A cohort study using the US Department of Veterans Affairs national databases involving 181,280 people with COVID-19, 4,118,441 contemporary controls and 4,286,911 historical controls of all ages found that COVID-19 survivors had an increased risk of diabetes beyond the first 30 days post infection [59].

### 5.1. Results of Meta-Analysis

The pooled relative risk estimates from a random-effects meta-analysis showed that the risk of new-onset T1D after COVID-19 increased by 62% (relative risk 1.622 (95% CI 1.347, 1.953)) (Figure 1).

Polling the individual estimates from all studies independent of the study design showed a 30% increased risk (relative risk 1.299 (95% CI 1.227, 1.375)) during the pandemic or after SARS-CoV-2 infection (Figure 1).

## 6. COVID-19 and Presymptomatic Type 1 Diabetes Autoimmunity

The association between infection with SARS-CoV-2 and the development of a presymptomatic T1D autoimmunity (stage 1 of T1D) has recently been investigated in an analysis of data from two large registries in Colorado, USA, and Bavaria, Germany [61].

The data basis was the Autoimmunity Screening for Kids (ASKs) study in Colorado (children and adolescents from 1 to 18 years) and the Bavarian Fr1da study from Germany (children and adolescents from 1 to 10.9 years). Children with antibodies against the receptor binding domain and nucleocapsid proteins of SARS-CoV-2 were considered COVID-19 positive. Indications of presymptomatic T1D autoimmunity (stage 1) were a detection of islet cell autoantibodies, insulin autoantibodies, autoantibodies against glutamate decarboxylase, tyrosine kinase IA-2, and against zinc transporter-8. Data from 51,970 children and adolescents could be analysed from 2020 to 2021. SARS-CoV-2 infection had been experienced by 1,524 children from the Colorado registry (32.3%) and 2,862 children (6.1%) from the Fr1da registry. The prevalence of multiple (Colorado 0.46% vs. 0.44%, Bavaria 0.28% vs. 0.31%) or singular high-affinity islet autoantibodies (Colorado 0.76% vs. 0.47%, Bavaria 0.14% vs. 0.11%) was not different between participants with and without antibodies against SARS-CoV-2 (combined adjusted odds ratio: multiple autoantibodies 1.06 (0.59–1.08), *p*=0.83; singular high-affinity islet autoantibodies: 1.34 (95% CI 0.70–2.44), *p*=0.36). Therefore, this study found no association between an infection with SARS-CoV-2 and the development of autoimmunity with regard to T1D. As a limitation of the study, it must be taken into account that the prevalence of islet cell or insulin autoantibodies was very low, which limits the power of the study. An important advantage of the study compared to many other studies mentioned is that a SARS-CoV-2 infection was defined by the detection of specific antibodies.

A study from Turin, Italy, investigated the infection status with SARS-CoV-2 in 39 children with newly diagnosed T1D between October 15, 2020, and April 15, 2021 [42]. 23% of these children had antibodies directed toward SARS-CoV-2, and 12% had a history of recent SARS-CoV-2 infection in themselves or in their family. Compared to the general paediatric population, the authors reported a 5.6-fold higher overall incidence rate of COVID-19 in the children with newly diagnosed T1D. The attributable risk for T1D of the pandemic cohort compared to the previous year was 44%. However, in relation to earlier years, the risk was smaller. The authors concluded that the imbalance of SARS-CoV-2 infection between children with T1D and the paediatric reference population may support a causal role for SARS-CoV-2 in triggering the autoimmune response underlying T1D. A major limitation of this single-centre study is the low number of participants and lack of seroprevalence data in the reference population. Therefore, nasal swab molecular analysis data were used as a reference in this study. In this context, it is important to note that data from Bavaria, Germany, in 2021 showed a prevalence of antibodies to SARS-CoV-2 in preschool and school children that was about three to four times higher than the cumulative reported virus-positive PCR results [60].

**Figure 2:**
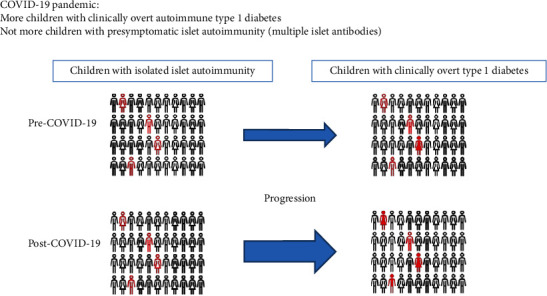
Schematic overview of suggested increased progression from presymptomatic islet autoimmunity to clinically manifest type 1 diabetes during the COVID-19 pandemic. Individuals with black border: no islet autoimmunity. Individuals with red border without filling: existing presymptomatic islet autoimmunity (also defined as stage 1 type 1 diabetes). Individuals with red filling: clinically overt type 1 diabetes (also defined as stage 3 type 1 diabetes). Please note that this is a simplified schematic representation. The frequencies of new cases of type 1 diabetes (red individuals) shown are not synonymous with the actual changes observed but are only meant to illustrate this.

In summary, there is an increase in T1D in children during the COVID-19 pandemic. The increase in the incidence rate of T1D concerns type 1a, i.e., with islet autoimmunity. However, the dynamics are surprisingly fast for an autoimmune process, with a latency period of only a few months. Whether this is a direct effect of SARS-CoV-2 itself or an indirect effect due to changes in the environment during the pandemic remains unclear. Many studies that suggest a direct impact of SARS-CoV-2 on the development of T1D have methodological weaknesses in in detecting SARS-CoV-2 infection or in defining COVID-19 cases, as the high proportion of asymptomatic children is not adequately taken into account. As no evidence of an increase in presymptomatic cases with isolated islet autoimmunity (also known as stage 1 of T1D) was found, albeit with limited power, this could also suggest an accelerated transition from presymptomatic patients with existing autoimmunity (stage 1) to clinically overt T1D (stage 3) (Figure 2).

## 7. COVID-19 Pandemic and Risk of Diabetic Ketoacidosis in New-Onset Type 1 Diabetes

Nationwide public health measures were rapidly implemented in response to the COVID-19 pandemic to contain the spread of SARS-CoV-2. These measures included social distancing, such as restricting social contacts, closing schools and advising people to stay at home. The number of admissions to health care facilities during the pandemic decreased significantly [62–64]. This has led to delays in diagnosis and some diseases have been diagnosed at an advanced stage [65–68]. For children with new-onset T1D, this delay in diagnosis led to an 85% increase in the odds of DKA during the first two months of the COVID-19 pandemic in Germany [69]. Ketoacidosis is an acute, life-threatening complication of missed early diagnosis of T1D [70–73]. The fact that the increased rate of DKA is caused by delayed diagnosis is shown by the fact that HbA1c levels at the time of diagnosis during the COVID-19 pandemic were higher than before [74]. An increase in the frequency of DKA in children and adolescents with new-onset T1D has been reported worldwide during the first months of the COVID-19 pandemic [25, 31–37, 40, 75–89].

A meta-analysis including 18 studies analysed the risk of DKA in patients with newly diagnosed T1D during the COVID-19 pandemic [22]. The cumulative risk showed a 44% (95% CI 26–65%) increased risk during the COVID-19 pandemic compared to before. A recent systematic review of factors associated with increased risk of DKA in paediatric patients with new-onset T1D, using a random-effects model from pooled data, found that presentation during the COVID-19 pandemic was associated with an odds ratio of 2.32 (95% CI 1.76, 3.06) for DKA [23].

A nationwide study in Germany investigated the rate of DKA in children and adolescents newly diagnosed with T1D on a month-by-month basis during the year 2020 [86]. Compared with the expected monthly ketoacidosis rates for 2020, the observed ketoacidosis rates in 2020 were significantly higher from April to September and again in December, with mean increases in these months ranging from 47% to 96%.

An international study of 13 diabetes registries found an increasing trend in DKA at diagnosis of T1D in children, which accelerated well before the emergence of COVID-19 and was then exacerbated by the pandemic [88]. This multicentre study found that the increased prevalence of DKA during the COVID-19 pandemic was mainly related to an increasing trend in DKA prevalence before the pandemic. These results suggest that the COVID-19 pandemic and the subsequent containment measures created ideal conditions for the emergence of preexisting problems related to the early diagnosis and care of children with new-onset T1D.

Furthermore, the increase in DKA during the COVID-19 pandemic was not a short-term phenomenon restricted to the initial lockdown. The adjusted observed frequencies of DKA at diagnosis of T1D were higher than predicted in both pandemic years: in 2020, 39.4% (95% CI 34.0–45.6%) vs. 32.5% (27.8, 37.9%) and in 2021, 38.9% (33.6–45.0%) vs 33.0% (28.3–38.5%). The adjusted absolute percentage increases were 6.9% (5.4–8.4%) for 2020 and 5.8% (4.4–7.3%) for 2021. Moreover, this study found that the prevalence of DKA was slightly associated with the pandemic containment measures. The stringency index was used to analyse the level of mitigation efforts. This index, scaled from 0 to 100, is an additive composite index that includes nine indicators: school closures, workplace closures, cancellation of public events, restrictions on public gatherings, closures of public transport, stay-at-home orders, public information campaigns, restrictions on internal movement, and controls on international travel [89]. Per ten-unit increase in the stringency index, the study found an estimated DKA risk ratio of 1.037 (95% 1.024–1.051) for 2020 and 1.028 (1.009–1.047) for 2021.

## 8. Conclusion

To date, there is no valid evidence that SARS-CoV-2 infections cause autoantibody-negative diabetes by direct *ß*-cell damage or autoantibody-positive T1D by triggering autoimmunity. Interventions to control the COVID-19 pandemic have led to a decrease in common paediatric viral infections, possibly reduced biodiversity of exposures, and increased psychological distress in children and adolescents. This may has subsequently led to an increased incidence rate of T1D, especially in the presence of pre-existing autoimmune prediabetes. Many studies suggesting a direct effect of SARS-CoV-2 have methodological weaknesses, because the high proportion of children asymptomatically infected with SARS-CoV-2 were not adequately taken into account. According to current knowledge; therefore, indirect effects of the COVID-19 pandemic are more likely to be associated with the observed increase in the incidence rate of T1D.

The development of the T1D incidence rate during the COVID-19 pandemic and the possible associations between SARS-CoV-2 infections and T1D risk should be investigated in long-term studies, especially in light of the emergence of new SARS-CoV-2 variants.

During the pandemic, there is a dramatic increase of the prevalence of DKA in children with newly diagnosed T1D. The massive increase in DKA cases is due to two multiplying factors during the pandemic: the increase in the number of children with new-onset T1D and the higher proportion of these children who have DKA. Timely access to healthcare, raising public and healthcare provider awareness of the symptoms of T1D through education and screening campaigns, and appropriate diabetes management during pandemics or in similar situations are key to preventing similar clusters of diabetic ketoacidosis in the future. Prevention campaigns have proven useful in reducing the prevalence rate of ketoacidosis in newly diagnosed children with T1D [90]. In addition, screening the population at risk for T1D either for *ß*-cell-antibodies or genetic risk, may identify children at risk and allow targeted observation to identify clinically over diabetes early, thus reducing the prevalence of DKA [91].

## Figures and Tables

**Table 1:** Relative increase of incident cases of type 1 diabetes (T1D) in the COVID-19 pandemic compared to prepandemic periods.

Study regions	Data sources	Age (years)	Prepandemic period		Pandemic period				
			Period	Observed	Period	Observed	Expected^1^	Observed vs. expected	*p*
				T1D cases		T1D cases	T1D cases	Relative risk (95% CI)^2^	
Rahmati et al. 2022 24 regions worldwide	24 studies, meta-analysis	Children	Periods in 2019 as in 2020 by study	2,706	Periods in 2020, varying by study	2,965	2,706	*1.083 (1.028, 1.142)* ^*3*^	*0.003* ^*3*^
Abdou 2022 Egypt, Cairo	1 paediatric centre	0.5–≤18	01.01.18–28.02.18	23	01.06.20–31.07.20 01.12.20–28.02.21	213	*58*	*3.672 (2.747, 4.909)*	*<0.001*
Alassaf 2022 Jordan, Amman	1 paediatric centre	Children	02.03.19−01.03.20	83	02.03.20−01.03.21	54	83	*0.651 (0.462, 0.917)*	*0.014*
Chambers 2022 USA, Phoenix	1 paediatric centre	<18	01.03.18–29.02.20	320	01.03.20–31.12.20	152	*133*	*1.143 (0.906, 1.442)*	*0.261*
Cherubini 2022 Italy	47 paediatric centres	<18	01.01.17–31.12.19	3,068	01.01.20–31.12.20	1,169	*1,023*	*1.143 (1.051, 1.243)*	*0.002*
Gottesman 2022 USA, San Diego	1 paediatric centre	<19	19.03.19–18.03.20	119	19.03.20–18.03.21	187	119	*1.571 (1.249, 1.977)*	*<0.001*
			19.03.17–18.03.20	10	01.07.20–31.07.20	15	10	*1.500 (0.674, 3.339)*	*0.321*
			19.03.17–18.03.20	10	01.02.20–28.02.21	21	10	*2.100 (0.989, 4.459)*	*0.053*
Kaya 2022 Turkey, Trabzon	1 paediatric centre	≤18	01.02.17–31.01.20	79	01.02.20–31.01.21	44	*26*	*1.692 (1.042, 2.748)*	*0.033*
Lee 2022 South Korea, Seoul	4 paediatric centres	<18	01.01.18–31.12.19	41	01.01.20–31.12.20	51	*21*	*2.429 (1.461, 4.037)*	*<0.001*
Leiva-Gea 2022 Spain	9 paediatric centres	≤18	01.01.15–31.12.19	1085	01.01.20–31.03.21	359	*271*	*1.325 (1.131, 1.551)*	*<0.001*
Luciano 2022 Brazil, Sao Paulo	1 paediatric centre	Children	01.04–31.08. In 2017–2019	25	01.04.20–31.08.20	18	*8*	*2.250 (0.978, 5.175)*	*0.056*
Mastromauro 2022 Italy, Chieti	1 paediatric centre	≤18	01.01.15–29.02.20	132	01.03.20–30.04.21	40	*30*	*1.333 (0.831, 2.141)*	*0.234*
Pillai 2022^5^ USA, Rhode Island	1 paediatric centre	Children	01.03–15.05. In 2018–2019	60	01.03.20–15.05.20	15	***30***	*0.500 (0.269, 0.929)*	*0.028*
Rusak 2022 Poland, Katowice	1 paediatric centre	Children	01.01.21–31.12.21	196	01.01.20–31.12.20	223	196	*1.138 (0.939, 1.378)*	*0.187*
Schiaffini 2022 Italy, Rome	1 paediatric centre	Children	01.01.17–31.12 19	290	01.01.20–31.12.21	220	*193*	*1.140 (0.940, 1.383)*	*0.184*
Wolf 2022 USA, 7 regions	7 paediatric centres	≤26	01.01.19–31.12.19	1,277	01.01.20–31.12.20	1,399	1,277	*1.096 (1.016, 1.182)*	*0.018*

Figures in italics were recalculated based on data reported in the original articles. ^1^Expected numbers of T1D cases for the pandemic period under study were estimated from observed numbers for the related prepandemic period considering the length of the prepandemic period. ^2^Estimated as ratio of observed and expected case numbers. ^3^Summary effect reported in the meta-analysis by Rahmati 2022.

**Table 2:** Relative increase of the incidence of type 1 diabetes (T1D) in the COVID-19 pandemic compared to prepandemic periods.

Study regions	Data sources	Age (years)	Prepandemic period		Pandemic period						
			Period	Observed	Period	Observed		Expected^1^		Observed vs expected	*P*
				T1D cases		T1D cases	Incidence (95% CI)^2^	T1D cases	Incidence (95% CI)^2^	Relative risk (95% CI)^3^	
Al-Abdulrazzaq 2022 Kuwait	Child diabetes registry	0.5–≤12	24.02.19–23.02.20	303	24.02.20 –23.02.21	324	40.2 (36.1, 44.8)	303	37.6 (33.6, 42.1)	*1.069 (0.914, 1.250)*	*0.403*
Cinek 2022 Czechia	Czech Childhood Diabetes Register	<15	01.01.10–31.12.19	—	01.01.20–31.12.21	956	27.2 (25.5, 29.0)	*824*	*23.4 (21.9, 25.1)*	1.160 (1.060, 1.280)	0.002
Denina 2022 Italy, Piedmont	1 pediatric centre	≤14	15.10.2016–15.04.2017 15.10.2017–15.04.2018 15.10.2018–15.04.2019	97	15.10.20 –15.04.21	39	*38.6 (28.2, 52.8)*	*24*	*22.5 (18.5, 27.5)*	*1.711 (1.180, 2.482)*	*0.005*
Dilek 2021 South Turkey, Adana	1 pediatric centre	<18	11.03.19–10.03.20	46	11.03.20 –11.03.21	74	*11.2 (8.9, 14.1)*	46	*6.9 (5.2, 9.2)*	*1.623 (1.123, 2.345)*	*0.010*
Guo 2022 USA, Florida	OneFlorida + network electronic health records	<18	01.01.17–31.03.20	1,921	01.04.20 –30.06.21	2,354	*32.6 (31.3, 33.9)*	1,921	*23.4 (22.7, 24.2)*	*1.392 (1.322, 1.465)*	*<0.001*
Hernandez-Herrero 2022 Spain, Tarragona	3 pediatric centres	<15	01.01.18–31.12.19	52	01.01.20 –31.12.20	37	*29.1 (21.1, 40.2)*	*26*	*20.4 (15.5, 26.8)*	*1.429 (0.937, 2.178)*	*0.097*
Ho 2021 Canada, Alberta	2 pediatric centres	<18	17.03.19–31.08.19	114	17.03.20 –31.08.20	107	*9.9 (8.2, 12.0)*	114	*10.6 (8.9, 12.8)*	*0.931 (0.715, 1.212)*	*0.596*
Kamrath 2022 Germany	German Diabetes Prospective Follow-up Registry	0.5–<18	01.01.11–31.12.19	—	01.01.20–30.06.21	5,162	24.4 (23.6, 25.2)	*4,489*	21.2 (20.5, 21.9)	*1.150 (1.100, 1.200)*	<0.001
		0.5–<6					18.6 (17.5, 19.8)		15.1 (14.1, 16.2)	1.230 (1.130–1.330)	<0.001
		6–<12					32.7 (31.2, 34.3)		27.7 (26.3, 29.2)	1.180 (1.110–1.260)	<0.001
		12– < 18					23.5 (22.2, 24.8)		22.2 (21.0, 23.5)	1.060 (0.980–1.130)	0.13
Mameli 2021 Italy, Lombardy	13 pediatric centres	≤17	01.01.19–31.12.19	231	01.01.20 –31.12.20	256	*16.0 (14.2, 18.1)*	231	*14.0 (12.9, 15.1)*	*1.143 (0.957, 1.365)*	*0.141*
McKeigue 2022 UK, scotland	Diabetes registry	≤14	01.01.15–31.12.21	—	01.01.20 –31.12.21	—	—	—	—	1.200	—
Pietropaolo 2022	TriNetX COVID-19 research network, electronic 60 healthcare organization	≤30	01.01.18–31.12.19	27,704	01.03.20–15.07.21	21,925	*30.1 (29.7, 30.5)*	*20,308*	*27.9 (27.6, 28.2)*	*1.080 (1.061, 1.099)*	*<0.001*
Pietrzak 2022 Poland	14 paediatric centres	≤18	15.03.19–14.03.20	1,391	15.03.20– 15.03.21	1,671	*25.9 (24.7, 27.2)*	1,391	*21.6 (20.4, 22.7)*	*1.202 (1.119, 1.290)*	*<0.001*
Salmi 2022 Finland	Helsinki University Hospital	≤15	01.04–31.10	231	01.04.20–31.10.20	84	56.0 (45.2, 69.3)	*58*	38.7 (34.0, 44.0)	*1.450 (1.130, 1.860)*	0.004
Shulman 2022^4^ Canada, Ontario	Health insurance data	1–17	01.01.17–29.02.20	—	01.03.20–30.09.21	1,570	*36.7 (35.0, 38.6)*	*1,440*	*33.7 (32.0, 35.5)*	*1.090 (0.910, 1.300)*	>0.05
VandenBoom 2022 Germany	IQVIA prescription database (LRx)	≤20	04.01.16−01.03.20	16293	02.03.20−02.01.22	7492	*26.7 (26.1, 27.3)*	5992	*21.2 (20.7, 21.8)*	*1.259 (1.217, 1.303)*	*<0.001*
			06–08	3,447	06.2020–08.2020	990	25.9 (24.1, 27.8)	689	18.0 (16.5, 19.6)	1.440 (1.130, 1.580)	*<0.001*
			In 2016–2019		05.2021–08.2021	1036	27.1 (25.3, 29.1)	629	16.5 (15.0, 18.0)	1.650 (1.490, 1.820)	*<0.001*
Vlad 2021^5^ Romania	Child diabetes registry	<15	01.01.19–31.12.19	—	01.01.20–31.12.20	*403*	*13.3 (12.1, 14.7)*	*347*	*11.4 (10.3, 12.7)*	*1.166 (1.010, 1.345)*	*0.036*

Figures in italics were recalculated based on data reported in the original articles. ^1^Expected incidences of T1D for the pandemic period under study were estimated from observed incidences for the related prepandemic period. ^2^Per 100,000 person-years. ^3^Estimated as ratio of observed and expected incidences. ^4^No differentiation between type 1 and type 2 diabetes (but 95% of children with diabetes in Ontario have type 1 diabetes). ^5^To estimate T1D case numbers, we used population data from EUROSTAT (https://ec.europa.eu/eurostat/databrowser/view/DEMO_PJANGROUP__custom_4092331/default/table?lang=en).

**Table 3:** Relative risk of type 1 diabetes after SARS-CoV-2 infection.

Study regions	Data sources	Age (years)	Pandemic period	(Pre-)pandemic control period	Cohort with documented SARS-CoV-2 infection		Cohort without documented SARS-CoV-2 infection or with other respiratory infection (RI)		With SARS-CoV-2 vs (without SARS-CoV-2 or with other RI)	*p*
					“Observed”		“Expected”		“Observed vs expected”	
					T1D cases	T1D incidence (95% CI)^1^	T1D cases	T1D incidence (95% CI)^1^	Relative risk 95% CI^2^	
Barret 2022^3^ USA	HealthVerity database	<18	01.03.20–28.06.21	01.03.20–28.06.21	1120^4^	399 (376, 423)^4^	853^5^	304 (284, 324)^5^	1.310 (1.200, 1.440)^7^	<0.05
	IQVIA database	<18	01.03.20–26.02.21	01.03.20–26.02.21	68^4^	316 (241, 391)^4^	132^5^	118 (98, 139)^5^	2.660 (1.980, 3.560)^*7*^	<0.05
				01.03.17–26.02.18			227^6^	126 (109, 142)^6^	2.160 (1.640, 2.860)^7^	<0.05

Kendall 2022	TriNetX analytics platform	≤18	01.03.20–31.12.21	01.03.20–31.12.21	56^8^	*19.6 (15.1, 25.5)* ^*8*^	30^8^	*10.5 (7.3, 15.0)* ^*8*^	1.960 (1.260, 3.060)^7^	<0.05
	Electronic health records				91^9^	*31.9 (25.9, 39.1)* ^*9*^	46^9^	*16.1 (12.1, 21.5)* ^*9*^	2.100 (1.480, 3.000)^7^	<0.05
	74 healthcare organization				123^10^	*43.1 (36.1, 51.4)* ^*10*^	72^10^	*25.2 (20.0, 31.8)* ^*10*^	1.830 (1.360, 2.440)^7^	<0.05

McKeigue 2022 UK, Scotland	COVID-19 database, diabetes registry	<35	01.03.20–22.11.21^11^	01.01.15–31.12.21^11^	31^12^	*29.0 (20.4, 41.3)* ^*12*^	1.005^13^	*9.1 (8.6, 9.7)* ^*13*^	*2.620 (1.810, 3.780)* ^*7*^	<0.001
					38^13^	*8.7 (6.3, 11.9)* ^*14*^			*0.860 (0.620, 1.210)* ^*7*^	0.4

Pietropaolo 2022	TriNetX COVID-19 research network, electronic medical records, 60 healthcare organization	≤18	01.01.20–11.06.21	01.01.20–11.06.21	26	*23.6 (16.1, 34.7)*	30^15^	*27.2 (19.0, 39.0)* ^*15*^	*0.867 (0.513, 1.466)* ^*16*^	0.595
		19–30			43	*26.3 (19.5, 35.4)*	23^15^	*14.0 (9.3, 21.1)* ^*15*^	1.869 (1.127, 3.106)^16^	0.014

Qeadan 2022 USA	CERNER REAL-WORLD-DATA electronic health records	<18	01.08.21–30.09.21^8^	01.08.21–30.09.21^8^	598^17^	*159.4 (147.1, 172.7)* ^*17*^	5376^18^	*98.7 (96.1, 101.3)* ^*18*^	*1.616 (1.485, 1.758)*	*<0.001*
		18–35			1003^17^	*181.4 (170.5, 193.0)* ^*17*^	9864^18^	*186.7 (183.0, 190.4)* ^*18*^	*0.972 (0.911, 1.037)*	*0.390*
		≥36			3304^17^	*232.8 (225.0, 240.9)* ^*17*^	18566^18^	*149.5 (147.4, 151.7)* ^*18*^	*1.557 (1.500, 1.616)*	*<0.001*
		All ages			5163^17^	*207.4 (201.8, 213.1)* ^*17*^	36348^18^	*146.5 (145.0, 148.1)* ^*18*^	*1.415 (1.375, 1.457)*	*<0.001*

Xie 2022	US Department of Veterans	All ages	01.03.20–30.09.21	01.03.20–30.09.21	49	28 (19, 41)	643^19^	17 (16, 19)^19^	*1.647 (1.029, 2.637)*	*<0.001*
USA	Affair national database			01.03.18–30.09.19			828^19^	22 (20, 23)^19^	*1.273 (0.808, 2.005)*	*0.101*

Figures in italics were recalculated based on data reported in the original articles. ^1^Per 100,000 person-years, if not stated otherwise. ^2^Estimated as ratio of the incidence in the cohort with documented SARS-CoV-2 infection and that in the cohort without documented SARS-CoV-2 infection, if not stated otherwise. ^3^No differentiation between type 1 and type 2 diabetes. ^4^Number of cases or incidence in the cohort with documented SARS-CoV-2 infection more than 30 days before diabetes diagnosis. ^5^Number of cases or incidence in the pandemic comparison cohort without documented SARS-CoV-2 infection. ^6^Number of cases or incidence in prepandemic comparison cohort with documented respiratory infection. ^7^Estimated hazard ratio. ^8^Number of cases or cumulative incidence with T1D diagnosis with 1 month after SARS-CoV-2 infection. ^9^Number of cases or cumulative incidence with T1D diagnosis with 3 month after SARS-CoV-2 infection. ^10^Number of cases or cumulative incidence with T1D diagnosis with 6 month after SARS-CoV-2 infection. ^11^Follow-up period. ^12^Number of cases or incidence per 100,000 person-days in the cohort with documented SARS-CoV-2 infection within 30 days before diabetes diagnosis. ^13^Number of cases or incidence per 100,000 person-days in comparison cohort without documented SARS-CoV-2 infection. ^14^Number of cases or incidence per 100,000 person-days in the comparison cohort with documented SARS-CoV-2 infection more than 30 days before diabetes diagnosis. ^15^Number of cases or incidence in the comparison cohort without documented SARS-CoV-2 infection. ^16^Estimated risk ratio from propensity score-matched analysis (reciprocal values of figures reported in Pietropaolo 2022). ^17^Number of cases or cumulative incidence in the cohort with documented SARS-CoV-2 infection. ^18^Number of cases or cumulative incidence in the cohort without documented SARS-CoV-2 infection. ^19^Number of cases or incidence in the cohort without documented SARS-CoV-2 infection.

## Data Availability

The data used to support the findings of the study are included within the article.
